# Clinical feasibility study of early 30-minute dynamic FDG-PET scanning protocol for patients with lung lesions

**DOI:** 10.1186/s40658-024-00625-3

**Published:** 2024-03-05

**Authors:** Fen Du, Xieraili Wumener, Yarong Zhang, Maoqun Zhang, Jiuhui Zhao, Jinpeng Zhou, Yiluo Li, Bin Huang, Rongliang Wu, Zeheng Xia, Zhiheng Yao, Tao Sun, Ying Liang

**Affiliations:** 1 Department of Nuclear Medicine, National Cancer Center/National Clinical Research Center for Cancer/Cancer Hospital & Shenzhen Hospital, Chinese Academy of Medical Sciences and Peking Union Medical College, Shenzhen, China; 2 Lauterbur Research Center for Biomedical Imaging, Shenzhen Institute of Advanced Technology, Chinese Academy of Sciences, Shenzhen, China

**Keywords:** Dynamic FDG-PET, Ki, Influx constant, Image quality, Diagnostic efficiency

## Abstract

**Purpose:**

This study aimed to evaluate the clinical feasibility of early 30-minute dynamic 2-deoxy-2-[^18^F]fluoro-D-glucose (^18^F-FDG) positron emission tomography (PET) scanning protocol for patients with lung lesions in comparison to the standard 65-minute dynamic FDG-PET scanning as a reference.

**Methods:**

Dynamic ^18^F-FDG PET images of 146 patients with 181 lung lesions (including 146 lesions confirmed by histology) were analyzed in this prospective study. Dynamic images were reconstructed into 28 frames with a specific temporal division protocol for the scan data acquired 65 min post-injection. Ki images and quantitative parameters Ki based on two different acquisition durations [the first 30 min (Ki-30 min) and 65 min (Ki-65 min)] were obtained by applying the irreversible two-tissue compartment model using in-house Matlab software. The two acquisition durations were compared for Ki image quality (including visual score analysis and number of lesions detected) and Ki value (including accuracy of Ki, the value of differential diagnosis of lung lesions and prediction of PD-L1 status) by Wilcoxon’s rank sum test, Spearman’s rank correlation analysis, receiver operating characteristic (ROC) curve, and the DeLong test. The significant testing level (alpha) was set to 0.05.

**Results:**

The quality of the Ki-30 min images was not significantly different from the Ki-65 min images based on visual score analysis (*P* > 0.05). In terms of Ki value, among 181 lesions, Ki-65 min was statistically higher than Ki-30 min (0.027 ± 0.017 ml/g/min vs. 0.026 ± 0.018 ml/g/min, *P* < 0.05), while a very high correlation was obtained between Ki-65 min and Ki-30 min (*r* = 0.977, *P* < 0.05). In the differential diagnosis of lung lesions, ROC analysis was performed on 146 histologically confirmed lesions, the area under the curve (AUC) of Ki-65 min, Ki-30 min, and SUVmax was 0.816, 0.816, and 0.709, respectively. According to the Delong test, no significant differences in the diagnostic accuracies were found between Ki-65 min and Ki-30 min (*P* > 0.05), while the diagnostic accuracies of Ki-65 min and Ki-30 min were both significantly higher than that of SUVmax (*P* < 0.05). In 73 (NSCLC) lesions with definite PD-L1 expression results, the Ki-65 min, Ki-30 min, and SUVmax in PD-L1 positivity were significantly higher than that in PD-L1 negativity (*P* < 0.05). And no significant differences in predicting PD-L1 positivity were found among Ki-65 min, Ki-30 min, and SUVmax (AUC = 0.704, 0.695, and 0.737, respectively, *P* > 0.05), according to the results of ROC analysis and Delong test.

**Conclusions:**

This study indicates that an early 30-minute dynamic FDG-PET acquisition appears to be sufficient to provide quantitative images with good-quality and accurate Ki values for the assessment of lung lesions and prediction of PD-L1 expression. Protocols with a shortened early 30-minute acquisition time may be considered for patients who have difficulty with prolonged acquisitions to improve the efficiency of clinical acquisitions.

## Background

2-deoxy-2-[^18^F]fluoro-D-glucose(^18^F-FDG) positron emission tomography (PET) /CT imaging has a great impact on the diagnostics and management of oncological patients and has gained tremendous use worldwide [1, 2]. Two ^18^F-FDG PET image acquisition frameworks are commonly used, namely static and dynamic acquisitions [3]. At present, static imaging is more widely used, and partly for convenience, the patients are scanned 60 min after administration of the tracer [4]. The standard uptake values (SUVs), especially maximum standard uptake value (SUVmax), and mean standard uptake value (SUVmean), represent semiquantitative measures of glucose uptake as the main quantitative indicators in clinical practice [5]. Although useful, SUVs are highly dependent on the interval between ^18^F-FDG injection and image acquisition. It can also be affected by blood glucose levels, and non-perfect injections [5, 6]. And SUVs have some limitations in a range of clinical tasks, including distinguishing between malignant vs. benign (e.g., inflammatory) uptake and assessment of treatment response [5, 7]. The need for an additional quantification to compensate for the shortcomings of semiquantitative assessment of PET is constantly increasing.

Dynamic ^18^F-FDG PET scanning has also been in use for a long time, which continuously acquires imaging data over usually 60 min. Compared with the single semi-quantitative SUVs provided by the commonly used static PET/CT, dynamic ^18^F-FDG PET can provide quantitative evaluation with kinetic rate constants, e.g., net influx rate Ki, tumor blood influx rate K1, tumor blood efflux rate k2, phosphorylation rate k3 [7–9]. Kinetic analysis of dynamic PET imaging with a more accurate assessment of changes in tumor metabolism has been shown to be effective in improving diagnostic accuracy and achieving appropriate therapy monitoring in many different types of cancer [10]. While comparing the rate constant values of K1, k2, and k3 estimated by ^18^F-FDG, Ki was more commonly used and had been shown to be useful for characterizing tumor metabolism and assessing therapy response by reflecting more accurate changes after therapy [11–15]. The Ki was superior to SUV in differential diagnosing solitary lung nodules and lymph nodes (LNs), as well as in delineating tumor volume [6–8, 16]. However, the long acquisition time, single bed-position dynamic acquisition (axial extent of view of 15-25 cm), invasive arterial blood sampling, large volume of post-processed data, and the relatively complex process limit the routine use of dynamic PET in clinical practice [5, 17–19]. With the update of new acquisition equipment (e.g., total-body PET) and the development of computational models, the problems of short acquisition fields, invasiveness, and data processing have basically been solved [9, 19], but shortening the acquisition time still requires further research.

A range of time windows involved in shortened dynamic ^18^F-FDG PET scanning protocols have been considered by several groups. Three scanning protocols are used in most studies: (i) only late-time dynamic scanning (such as 30-60 min post-injection), which lacks early-time data. It often needs another plasma input function (IF), such as population-based IF (PBIF). However, it may introduce errors into the parametric analysis as it does not take into account the specificity of the individual IF [20]; (ii) two-short-dynamic-scanning, which requires two separate scans after ^18^F-FDG injection, such as (0-6 min + 60-75 min) scans, (0-10 min + 55-60 min) scans, (0-10 min + 40-60 min) scan and two 5-min scans [21–25]. These studies showed that the generated parameter metabolic rate of glucose (MR_glu_) [21] or Ki [22, 24, 25] is highly correlated with the reference parameter calculated from the full scan. However, such a protocol requires a break in the middle, and the problem of image registration can limit the feasibility; and (iii) only early-time scanning, with dynamic analysis carried out based on the acquired data from the early frames, such as 0-30 min scans. The use of early dynamic PET data to calculate Ki is based on the hypothesis that the Patlak plot, which represents the metabolic rate of MR_glu_ in lesions, enters a linear phase at an early stage for which the slope can theoretically be extracted. During this period, the dynamic trend can be measured and is consistent with that provided by a standard scan [26]. These studies published by Torizuka et al. [7] and Visser et al. [27] showed that the MR_glu_ values or Ki derived from the standardized scans and the 30 min scans had strong correlation, suggesting that a shorter imaging duration of 0-30 min may represent a clinically viable alternative to an imaging sequence of 0-60 min for kinetic modeling of FDG-PET. The early-time scanning protocol also had the advantage of providing accurate individual PBIF estimates, avoiding the problem of inaccurate image registration, and more accessible for patients. Although some studies have shown a strong correlation between the dynamic parameter Ki obtained with a shortened acquisition time of 0-30 min and that obtained with 60 min, these are small sample size studies and the consistency of the diagnostic results for benign and malignant lesions has not been investigated. Simplified acquisition protocols always involve a trade-off between clinical convenience and quantitative accuracy. In balancing these competing considerations, how the protocol is adapted depends on the clinical study or diagnostic application [17].

The development and clinical use of immune checkpoint inhibitors (ICIs) in recent years has opened new frontiers in the treatment of non-small cell lung cancer (NSCLC) [28]. National Comprehensive Cancer Network (NCCN) guidelines recommend treatment based on expression of programmed death-ligand 1 (PD-L1) in tumor as determined by immunohistochemistry (IHC) [29]. PD-L1 positivity was associated with significantly higher objective response rate, longer progression-free survival (PFS), and longer overall survival (OS) [30]. However, for patients who are unable to provide histological samples or who fail IHC testing, an alternative non-invasive method of measuring PD-L1 status would have important implications for clinical decision support. Some studies have demonstrated that the predictive value of SUVmax on FDG PET/CT in PD-L1 expression from the lung cancer patients at the initial diagnosis [31–34]. But data on the application of dynamic PET/CT in immunotherapy are limited, e.g., the predictive value of Ki in PD-L1 expression.

Accordingly, our study intends to further investigate the clinical feasibility of early 30-minute dynamic PET in terms of image quality, consistency of quantitative parameters, diagnostic efficacy in benign and malignant lung lesions, and predictive value in PD-L1 expression.

## Materials and methods

### Patient demographics

This prospective study was approved by the ethics committee of Cancer Hospital & Shenzhen Hospital, Chinese Academy of Medical Sciences (Clinical Trial Number: KYLH2022-1), which followed the 1964 Helsinki Declaration ethical standards and its subsequent amendments. All patients were provided written informed consent. Dynamic FDG-PET scans of the chest region were performed on 229 patients with clinical suspicion of lung cancer without treatment from May 2021 to September 2023. The following criteria were used to determine inclusion: (i) successful completion of dynamic FDG-PET, breath-holding chest CT, and whole-body static PET/CT scan; (ii) lung lesions with FDG avid confidentially identified by the readers. The exclusion criteria were as follows: patients failed to obtain histological confirmation of at least one lung lesion within two weeks (by biopsy or surgery). Finally, 146 patients were enrolled, including 87 males and 59 females, with age of 59.71 ± 10.75 (35–81) years. In these 146 patients, a total of 181 FDG avid lung lesions that were completely located in the field-of-view of the scanner were identified, of which 36 have maximum diameter (d_max_) < 1.5 cm, 77 have d_max_≥1.5 cm and < 3.0 cm, 68 have d_max_≥3.0 cm. Out of 181 lung lesions, 146 lesions were histologically confirmed by biopsy or surgery (each patient had one lesion), including 128 malignant and 18 benign lesions. Among 128 malignant lesions, 73 NSCLC lesions had definite PD-L1 expression results. PD-L1 expression was positive in 39 lesions and negative in 34 lesions. The characteristics of the patients and lung lesions are shown in Table 1.

**Table 1:** Characteristics of the patients and lung lesions

Characteristic	Distribution
**Number of patients**	**146**
**Sex (Male/Female, n)**	**87/59**
**Age (mean ± SD, range, years)**	**59.71 ± 10.75 (35–81)**
**Number of lung lesions**	**181**
With pathology	146 (80.66%)
Without pathology	35 (19.34%)
**Diameter, n**	
d_max_<1.5 cm	36 (19.89%)
1.5 cm ≤ d_max_<3.0 cm	77 (42.54%)
d_max_≥3.0 cm	68 (37.57%)
**SUVmax of lung lesions (mean ± SD, range)**	**11.03 ± 6.53 (1.10–47.50)**
With pathology	11.90 ± 6.57 (1.10–47.50)
Without pathology	7.40 ± 4.72 (1.90–19.50)
PD-L1 positive	14.80 ± 7.32 (3.50–39.20)
PD-L1 negative	9.05 ± 6.31 (1.90–27.00)
**Pathological types of lung lesions, n**	**146**
Malignant	128
AC	94 (64.38%)
SCC	15 (10.27%)
SCLC	7 (4.79%)
Other primary lung malignant tumors	9 (6.16%)
Pulmonary metastasis	3 (2.05%)
Benign	18
Pulmonary hamartoma	1 (0.68%)
Pulmonary sequestration	1 (0.68%)
Tuberculosis	2 (1.37%)
Nonspecific inflammation	14 (9.59%)
**PD-L1 expression in NSCLC, n**	**73**
PD-L1 positivity	39 (53.42%)
AC	34 (46.57%)
SCC	3 (4.11%)
Others	2 (2.74%)
PD-L1 negativity	34 (46.58%)
AC	31 (42.47%)
SCC	3 (4.11%)

AC, SCC, SCLC, NSCLC, PD-L1 and d_max_ represent adenocarcinoma, squamous cell carcinoma, small cell lung cancer and non-small cell lung cancer, programmed death-ligand 1and maximum diameter, respectively

### Dynamic FDG-PET acquisition and reconstruction

All patients avoided strenuous exercise for 24 h and fasted for at least 6 h prior to the PET/CT scan (Discovery MI PET/CT, GE Healthcare, Milwaukee, USA). At the time of ^18^F-FDG injection, blood glucose was below 8.0mmol/L. Firstly, the breath-holding chest CT and whole-body CT scans (from the head to the mid-femur in the supine position with the arms raised) were performed using the following parameters: tube voltage, 120 kV; tube current, 10-220 mA; pitch, 1.375:1; noise index, 20. Then, the dynamic PET scans of the chest region (encompassing a 20-cm axial field of view) were obtained immediately after the injection of ^18^F-FDG (mean ± SD, 288.78 ± 49.69MBq, range, 203.15-423.65MBq) from an intravenous indwelling needle. The total dynamic scan lasted for 65 min, and its data were partitioned into 28 frames as follows: 6 × 10s, 4 × 30s, 4 × 60s, 4 × 120s, 10 × 300s. The 21st frame represents the last frame of the first 30 min of data. Lastly, a whole-body static PET scan was performed with the speed of 1.5 min/bed at the end of the dynamic acquisition. The attenuation correction was performed using CT data, and PET image reconstruction was performed using the block sequential regularized expectation maximization (BSREM) reconstruction algorithm with 25 iterations and 2 subsets, matrix size, 256 × 256.

### PET/CT data analysis

#### Dynamic parameter Ki analysis

The dynamic parameters Ki of the first 30 min post-injection (Ki-30 min) and the first 65 min post-injection (Ki-65 min) were obtained based on the two-tissue irreversible compartment model by using the imaging frames for the first 30 min and the first 65 min post-injection, respectively. The image-derived input function (IDIF) was extracted from the ascending aorta by drawing a 10-mm-diameter region-of-interest on six consecutive slices in an image obtained by combining early time frames (0-60s), where the effects of motion and partial volume were less prominent than in the left ventricle. The uptake difference in blood and plasma was not accounted for. In this model, we assumed unidirectional uptake of ^18^F-FDG (i.e., k4 = 0), with irreversible trapping in tissue as ^18^F-FDG-6-PO [35]. Ki-65 min and Ki-30 min parametric images of each dynamic scan were generated using voxel-based analysis. Given the large number of voxels in a PET image, the Lawson-Hanson non-negative least squares algorithm was applied to solve a linearized problem instead of the conventional nonlinear one [36]. The 3D volume of interest (VOI) of each lesion in the SUV images of 65 min and 30 min were delineated, respectively, using semi-automatic methods with a threshold of 40% SUVmax in the ITK-snap software (version 4.9). For lesions with physiological uptake in the periphery tissue, two experienced nuclear medicine physicians manually delineated 3D VOIs on a slice-by-slice basis. The segmented VOI was then applied to the Ki images of 65 min and 30 min, respectively, to extract quantitative measurements from each scan.

#### Visual quality assessment and lesion detectability of Ki images

The Ki images were independently evaluated by two clinical nuclear medicine experts with more than 3 years of experience. The Ki images of 65-min and 30-min scans of each subject were anonymized and presented in random order to them. According to the Likert quintile [37], the subjective scores ranging from 1 to 5 of 5 categories were as follows: artifact reduction (ranging from 1 [enormous image artifact] to 5 [no image artifact]), noise suppression (ranging from 1 [enormous image noise] to 5 [no perceivable image noise]), contrast retention (ranging from 1 [hard to distinguish lesion edge] to 5 [very sharp lesion edge]), lesion discrimination (ranging from 1 [difficulty in lesion detection] to 5 [high confidence for small, low uptake lesion]), overall image quality (ranging from 1 [poor overall image quality] to 5 [excellent overall image quality]). Image quality scores of 3 or higher were qualified, indicating that the needs of clinical diagnosis could be met, whereas image quality scores of 1–2 did not meet the needs of clinical diagnosis [38, 39]. The final score is the average score from two clinical nuclear medicine experts.

All FDG avid lesions confidentially identified in Ki images by the readers were counted and the maximum diameter (d_max_) of the lesion was measured in breath-holding chest CT. The SUVmax of all FDG-avid lesions in standard static PET/CT were recorded. In the case of peripheral lung lesions combined with obstructive pneumonia or atelectasis, the d_max_ of the lesion was measured by delineating the lesion on the static PET image with a threshold of 40% SUVmax. The partial-volume effect (PVE) could introduce large quantitative bias, especially in lesions with diameters less than 3 times the resolution of the imaging system [40]. Therefore, the lung lesions were classified into the group of d_max_<1.5 cm, 1.5 ≤ d_max_<3.0 cm, d_max_≥3 cm based on the spatial resolution of the PET/CT system used in this study of 0.5 cm. The result of 65 min’s scan served as the reference to test the lesion detectability. The lesion detectability was determined by assessing the lesion detection rate of the lesion in this study.

### Pathological evaluation

Biopsy or surgical specimens were reviewed by two independent pathologists with more than 10 years of experience in lung cancer pathology. All specimens were sectioned and examined conventionally using hematoxylin-eosin staining. IHC staining was also performed at the pathologist’s discretion. The platform of Ventana BenchMark ULTRA and the antibody of Dako 22C3 were used for PD-L1 staining to quantify the presence of PD-L1. Tumor proportion score (TPS) was recorded as the percentage of PD-L1 positive tumor cells over all tumor cells, and TPS ≥ 1% were considered PD-L1 positive expression [34].

### Statistical analysis

MedCalc 20.010 software (MedCalc Software Ltd) was used for statistical analysis. Continuous group data were all non-normally distributed by Kolmogorov-Smirnov test and presented as mean ± SD as appropriate. The percentage difference (D%) in Ki-65 min and Ki-30 min were calculated (D%=absolute value of difference of Ki-65 min and Ki-30 min divided by the Ki-65 min) [7]. The differences in subjective scores of Ki image quality, Ki value, and D% with different groups were compared by the Wilcoxon rank-sum test. Spearman’s rank correlation coefficient was used to assess the correlation between Ki-30 min and Ki-65 min. Receiver operating characteristic (ROC) curve analyses were performed to evaluate the diagnostic accuracy of Ki-30 min, Ki-65 min and SUVmax in differentiating benign and malignant lung lesions and predicting PD-L1 expression. The differences in area under the curve (AUC) were determined by Delong’s test. The significant testing level (alpha) was set to 0.05.

## Results

### Comparison of visual quality assessment and lesion detectability of Ki images

**Fig. 1:**
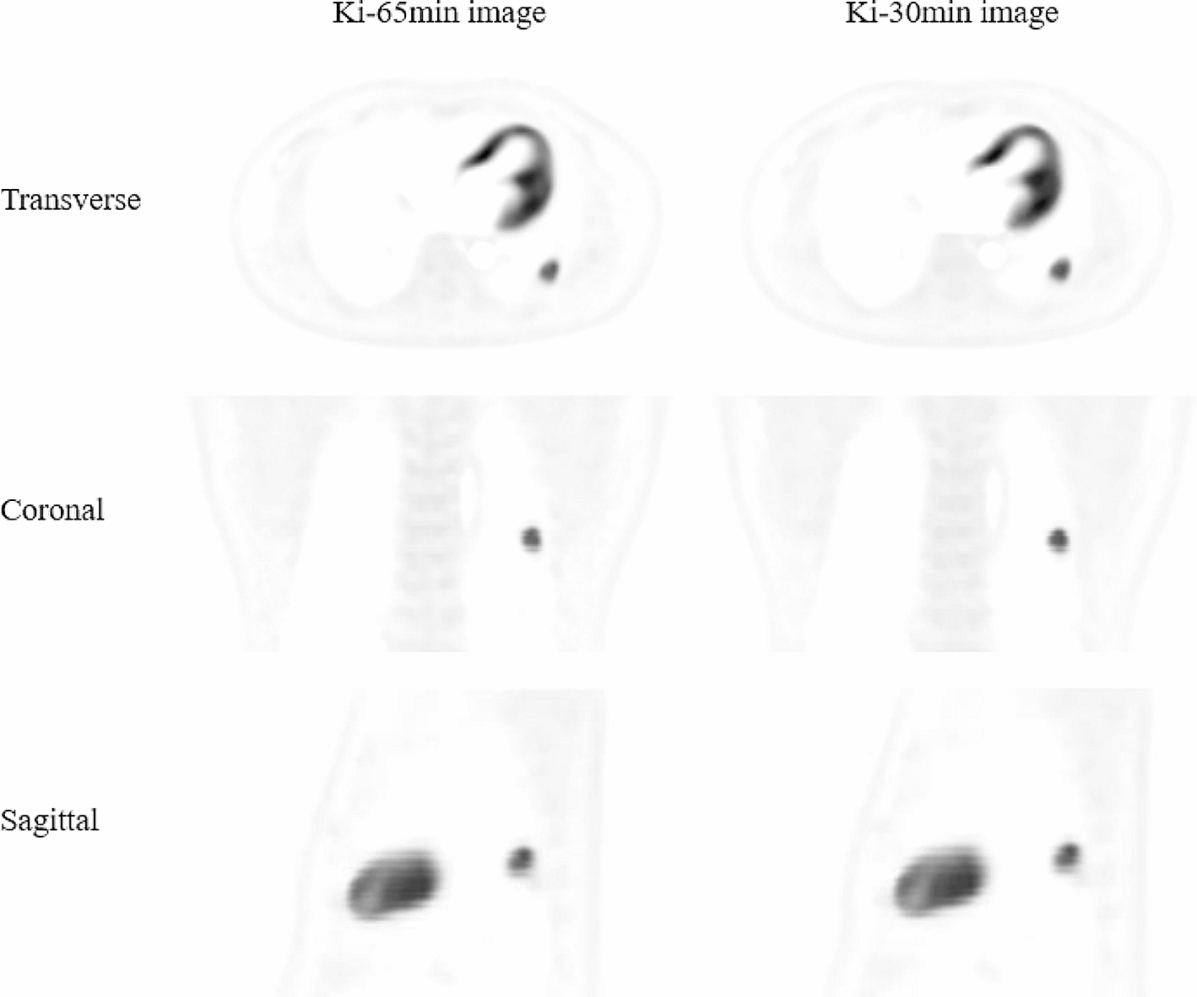
Representative Ki-65 min images and Ki-30 min images at different planes (the transverse, coronal and sagittal plane). Ki-65 min images and Ki-30 min images were found with good quality and showed no visual distinction between the two

All the image quality scores were above 3 in terms of five categories (artifact reduction, noise suppression, contrast retention, lesion discrimination, and overall quality) that were determined to meet clinical needs. Table 2 presents the subjective scores in five categories of the Ki-30 min images and the Ki-65 min images. No significant differences were found for different aspects of image quality between the Ki-30 min images and the Ki-65 min images (*P* > 0.05) (Fig. 1).

In terms of lesion detectability, 181 lung lesions were identified in both Ki-65 min and Ki-30 min images.

**Table 2:** Quantitative subjective scores in terms of five categories of the Ki-30 min images and the Ki-65 min images

Categories	Ki-65 min images	Ki-30 min images	Z value	*P* value
Artifact reduction	4.13 ± 0.48	4.11 ± 0.49	-1.51	0.13
Noise suppression	3.81 ± 0.52	3.79 ± 0.52	-1.52	0.13
Contrast retention	4.24 ± 0.58	4.21 ± 0.57	-1.26	0.21
Lesion discrimination	4.38 ± 0.52	4.36 ± 0.53	-0.78	0.44
Overall image quality	4.16 ± 0.53	4.14 ± 0.55	-1.49	0.14

### Assessment of quantitative dynamic parameter Ki

**Fig. 2:**
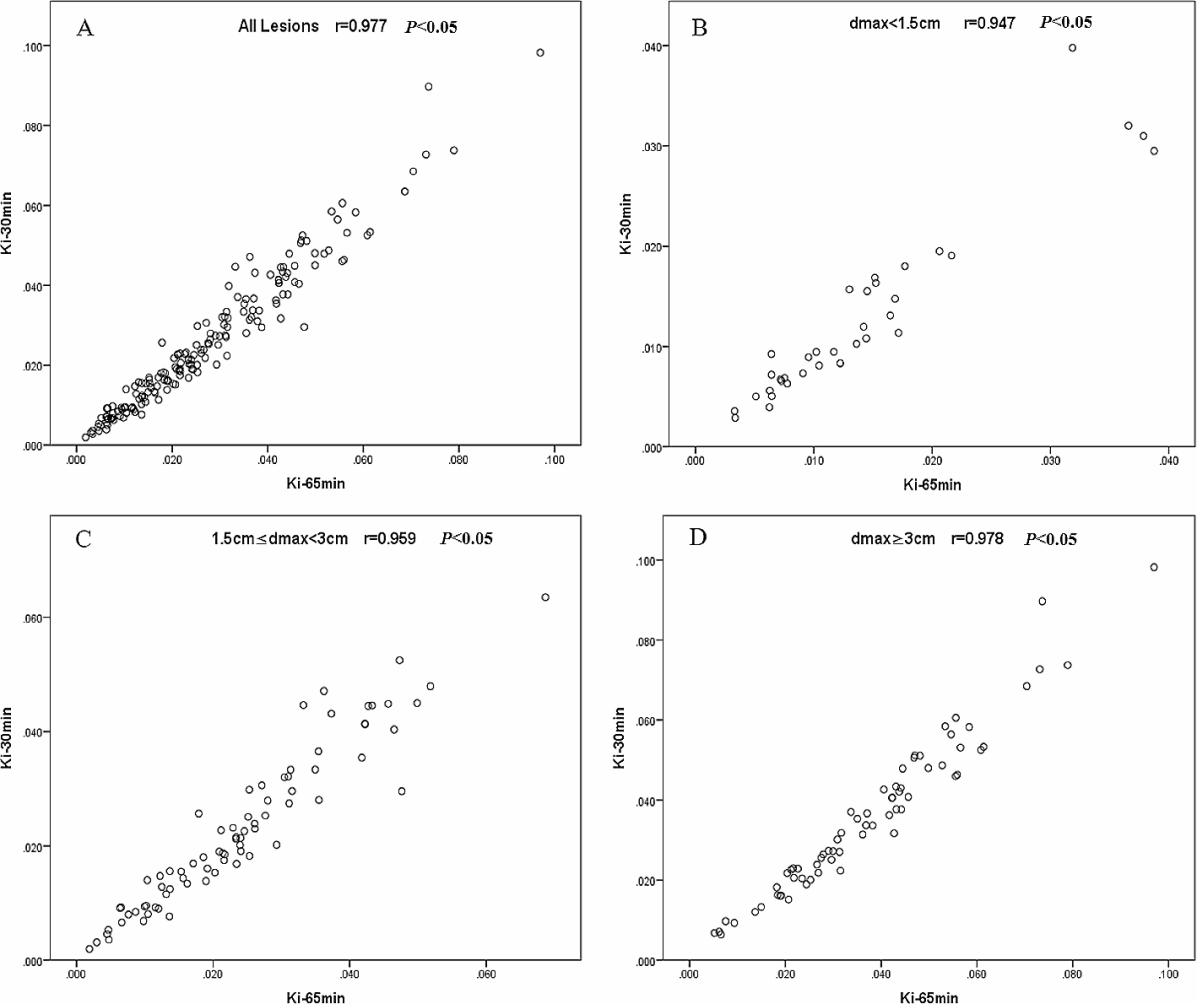
Correlations in Ki-65 min and Ki-30 min in all lesions(**A**), the groups of d_max_<1.5 cm (**B**), 1.5 cm ≤ d_max_<3.0 cm(**C**), and d_max_≥3.0 cm (**D**)

Among 181 lesions, there were very high correlations in Ki-65 min and Ki-30 min (*r* = 0.977, *P* < 0.05) (Fig. 2A). Figure 2B-D also show that there was a very high correlation of Ki-65 min and Ki-30 min within the group of d_max_<1.5 cm (*r* = 0.947, *P* < 0.05), 1.5 cm ≤ d_max_<3.0 cm (*r* = 0.959, *P* < 0.05), and d_max_≥3.0 cm (*r* = 0.978, *P* < 0.05).

The mean ± SD Ki-65 min and Ki-30 min of 181 lesions were 0.027 ± 0.017 ml/g/min and 0.026 ± 0.018 ml/g/min, respectively, and the difference was statistically significant (*P* < 0.05). The differences between Ki-65 min and Ki-30 min in the group of d_max_<1.5 cm (0.014 ± 0.009 ml/g/min vs. 0.013 ± 0.009 ml/g/min), 1.5 cm ≤ d_max_<3.0 cm (0.024 ± 0.014 ml/g/min vs. 0.023 ± 0.014 ml/g/min), and d_max_≥3.0 cm (0.037 ± 0.019 ml/g/min vs. 0.035 ± 0.020 ml/g/min) were statistically significant (*P* < 0.05).

The mean ± SD D% of the 181 lesions was 13.03%±10.38% (0.16-44.87%), and that in the groups of d_max_<1.5 cm, 1.5 cm ≤ d_max_<3.0 cm, and d_max_≥3.0 cm were 16.95%±10.29% (1.69-44.87%), 13.93%±11.70% (0.16-44.20%), and 9.94%±7.75% (0.21-31.70%), respectively. D% in the group of d_max_≥3.0 cm was significantly lower than that of d_max_<1.5 cm and 1.5 cm ≤ d_max_<3.0 cm (*P* < 0.05), while no significant difference was found between the groups of d_max_<1.5 cm and 1.5 cm ≤ d_max_<3.0 cm (*P* > 0.05).

### The diagnostic performance of quantitative dynamic parameter Ki

**Fig. 3:**
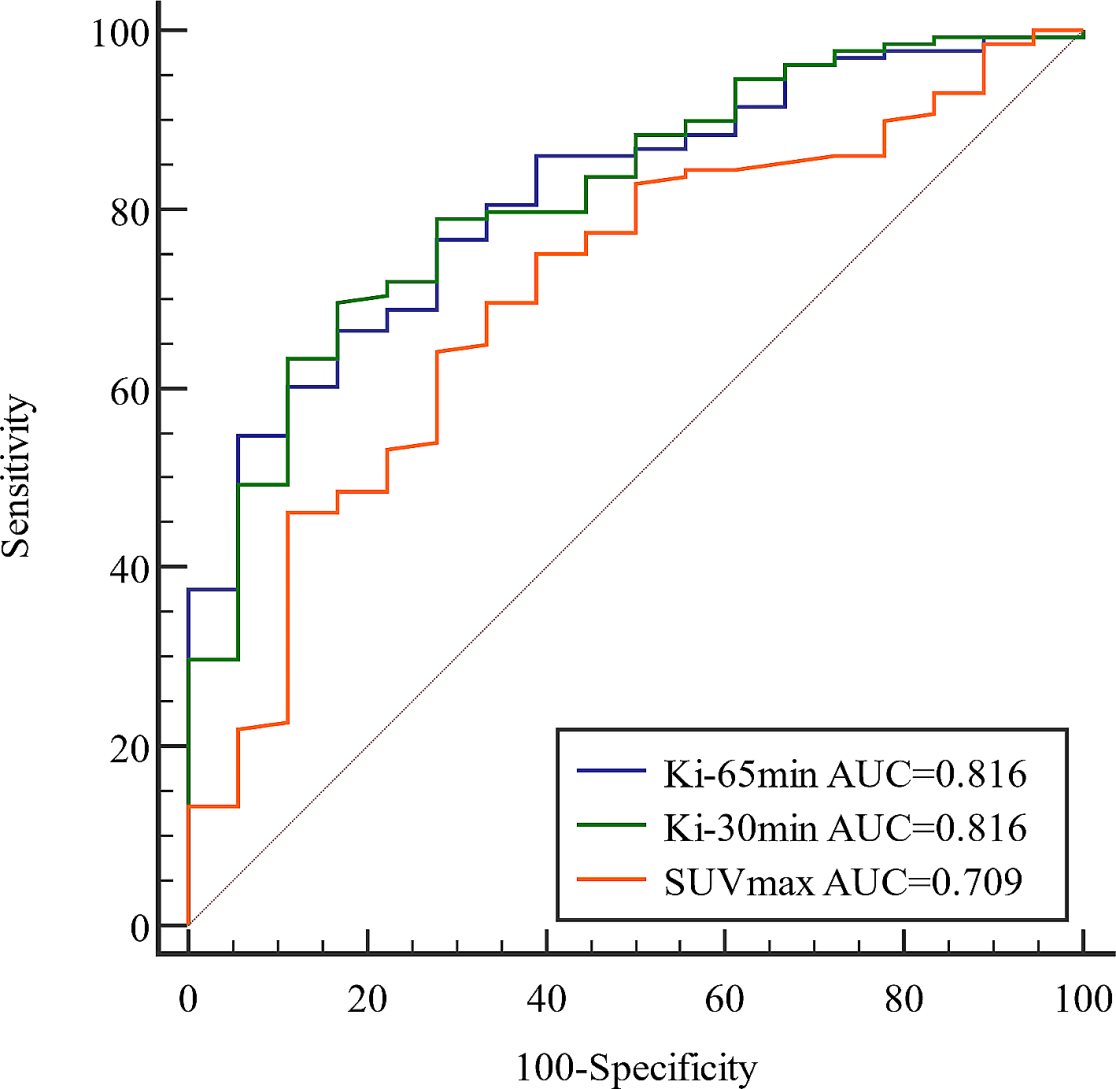
The ROC curve of Ki-65 min, Ki-30 min and SUVmax in the differential diagnosis of malignant and benign lung lesions

The ROC curves were plotted in 146 histologically confirmed lung lesions to determine the diagnostic accuracy of Ki-65 min, Ki-30 min and SUVmax in differentiating between benign and malignant lesions (Fig. 3). The optimal cut-off value of Ki-65 min was 0.022 ml/g/min, with an AUC of 0.816 (95%*CI*:0.744–0.876), a sensitivity of 66.40%, and a specificity of 83.30%. The optimal cut-off value of Ki-30 min was 0.018 ml/g/min, with an AUC of 0.816 (95%*CI*:0.743–0.875), a sensitivity of 69.50%, and a specificity of 83.30%. The optimal cut-off value of SUVmax was 9.65, with an AUC of 0.709 (95%*CI*:0.628–0.781), a sensitivity of 64.10%, and a specificity of 72.20%. And when the cut-off value of SUVmax was 2.50, the sensitivity and specificity were 98.40% and 5.6%, respectively. According to the results of the Delong test, no significant difference in the diagnostic accuracy was found between Ki-65 min and Ki-30 min (*P* > 0.05), while the diagnostic accuracies of Ki-65 min and Ki-30 min were both significantly higher than that of SUVmax (*P* < 0.05).

### The relationship between quantitative dynamic parameter Ki and PD-L1 expression

**Fig. 4:**
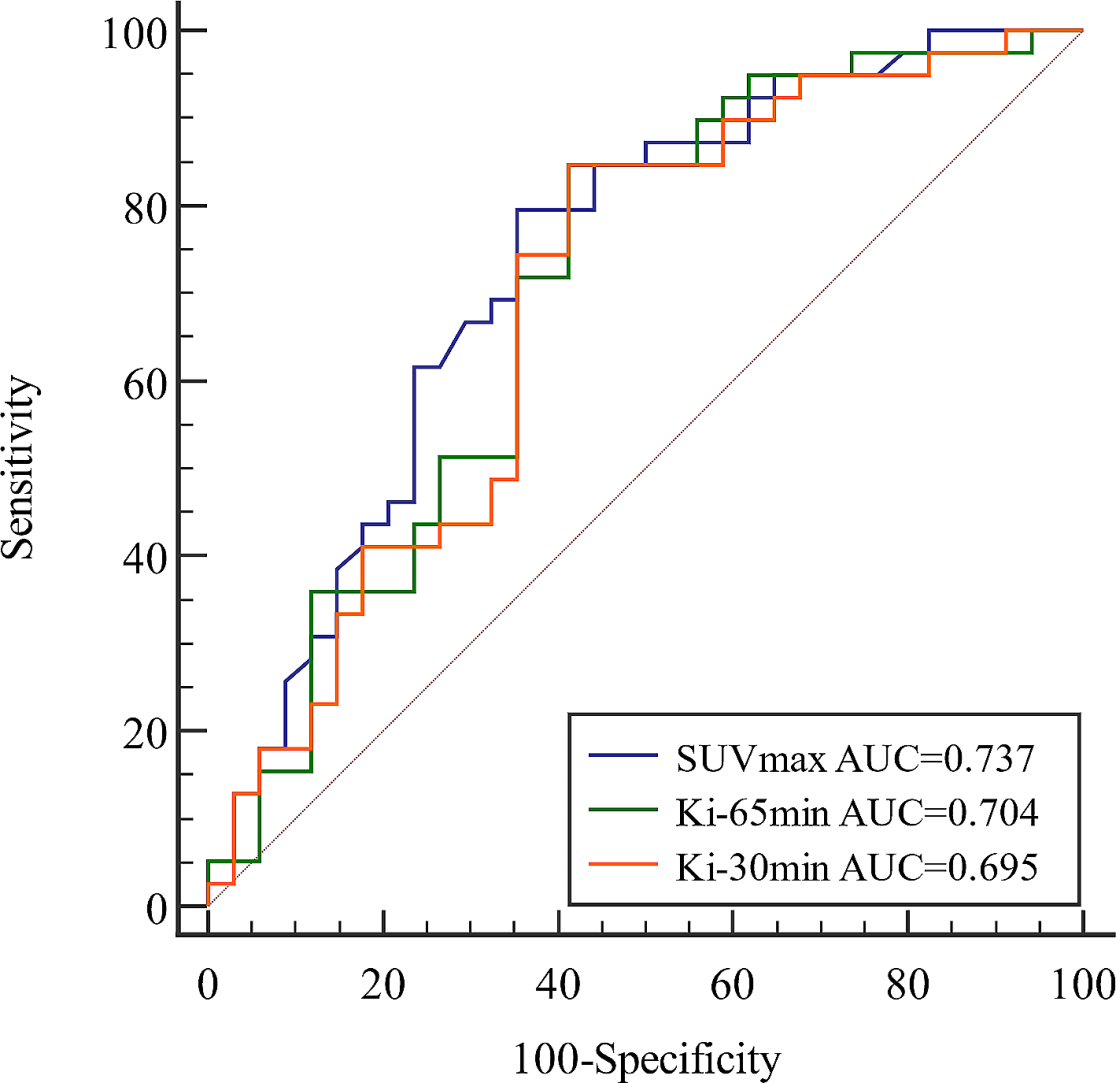
The ROC curve of Ki-65 min, Ki-30 min and SUVmax in predicting PD-L1 positive NSCLC lesions

In 73 NSCLC lesions with definite PD-L1 expression results, the Ki-65 min (0.035 ± 0.018 ml/g/min vs. 0.024 ± 0.018 ml/g/min, *P* < 0.05), Ki-30 min (0.034 ± 0.019 ml/g/min vs. 0.023 ± 0.019 ml/g/min, *P* < 0.05), and SUVmax (14.80 ± 7.32 vs. 9.05 ± 6.31, *P* < 0.05) in PD-L1 positive lesions were significantly higher than that in PD-L1 negative lesions. The ROC curves were plotted to determine the value of Ki-65 min, Ki-30 min, and SUVmax in predicting PD-L1 positive NSCLC lesions (Fig. 4). The optimal cut-off value of Ki-65 min, Ki-30 min, and SUVmax was 0.020 ml/g/min (AUC of 0.704, sensitivity of 84.60%, and specificity of 58.80%), 0.018 ml/g/min (AUC of 0.695, sensitivity of 84.60%, and specificity of 58.80%), and 9.55 (AUC of 0.737, sensitivity of 79.50%, and specificity of 64.70%), respectively. No significant differences in predicting PD-L1 positive NSCLC lesions were found among Ki-65 min, Ki-30 min, and SUVmax (*P* > 0.05), according to the results of the Delong test.

## Discussion

The main challenge of dynamic FDG-PET imaging to achieve clinical translation is partly due to the long scanning time [5, 41]. Optimal acquisition times for various applications remain to be determined. In this study of patients with lung lesions, we evaluated the clinical feasibility of shortening the dynamic acquisition time from 65 min to 30 min by comparing the image quality and quantitative dynamic parameters Ki of 6-5 min and 30-minute dynamic acquisitions. Our results showed that Ki-65 min images and Ki-30 min images both have good visual quality in terms of artifact reduction, noise suppression, contrast retention, lesion discrimination, and overall quality. Quantitative analyses showed that Ki-65 min and Ki-30 min were very highly correlated, and had similar values in differentiating benign and malignant lung lesions and predicting PD-L1 positive NSCLC lesions.

In terms of image quality, our study found that the quality of Ki-30 min images is as good as that of Ki-65 min images by visual quality assessment. In a different setting by Wang et al., they compared the MR_glu_ images of two-short-dynamic-scanning protocols (0-6 min + 60-75 min) and standard scanning protocols concluding that both protocols produced good-quality MR_glu_ images with no visual distinction [21]. In addition, our study found that the number of lesions detected in the two image types is consistent. This indicated that the Ki-30 min images will be able to meet the clinical requirements and early 30-minute dynamic FDG-PET acquisition might have the potential to improve clinical workflow.

In addition, regarding the assessment of the quantitative parameter Ki, our study showed a significant correlation with moderate variability between Ki-30 min and Ki-65 min (*r* = 0.977, D%=13.03%±10.38%), which was consistent with the study of 20 patients with untreated primary lung cancer published by Torizuka et al. (*r* = 0.966, D%=11.42%±11.31%) [7]. The same results were also found in the study that concluded 35 lesions of 15 different tumor patients by Chen et al. [26], which showed that Ki-30 min and Ki-60 min have an excellent agreement with *r* = 0.987. These results suggested that a 30-min shortened dynamic acquisition is sufficient to calculate Ki values comparable to 60-min dynamic acquisitions with clinical feasibility. On the other hand, our results also showed that the value of Ki-65 min is significantly higher than the Ki-30 min (0.027 ± 0.017 ml/g/min vs. 0.026 ± 0.018 ml/g/min, *P* < 0.05). The average uptake values within tumor isocontours contain statistical noise, which is reflected in uncertainties in the slope, and accordingly in the Ki values [27]. This might partly explain the difference between Ki-65 min and Ki-30 min.

**Fig. 5:**
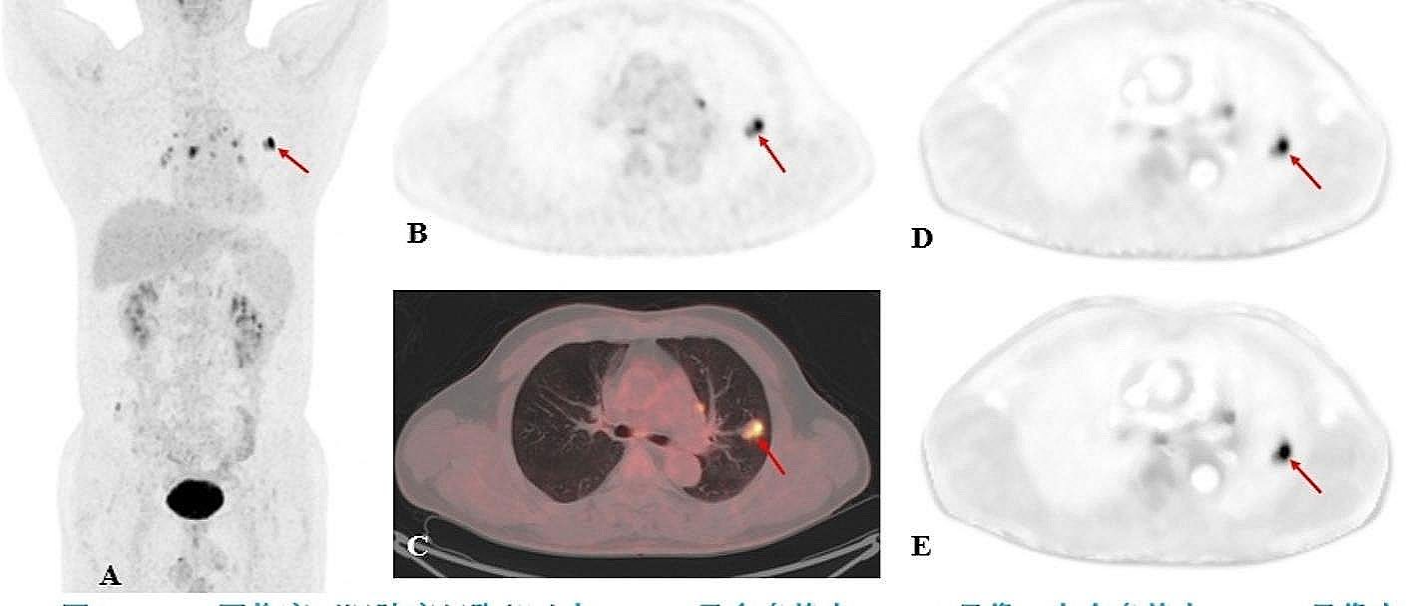
A 60-year-old male patient with clinical suspicion of lung cancer. PET/CT scan showed FDG-avid pulmonary nodules in the upper lobe of the left lung(red arrow), with a size of 2.2 × 1.3 cm, SUVmax of 12.2, Ki-65 min of 0.016 ml/g/min, and Ki-30 min of 0.014 ml/g/min. Surgical pathology confirmed a granulomatous lesion. In addition, PET/CT scan showed many FDG-avid LNs in the mediastinum. (**A**, static PET maximum intensity projection; **B**, static PET SUV image; **C**, static PET/CT fusion image; **D**, dynamic PET Ki-65 min image; **E**, dynamic PET Ki-30 min image)

In terms of diagnostic efficacy, the previous study performed by Skawran et al. in 60 cancer lesions and 17 inflammatory/infectious lesions has shown that using a cut-off value for Kimax-60 min of 0.026 ml/g/min delivers a sensitivity of 63.3% and a specificity of 82% for the detection of cancer lesions [42]. And previous research by our team also found that a cut-off value of Ki-65 min of 0.022 ml/g/min was identified as the optimal compromise point between sensitivity and specificity, with values of 39.50% and 91.80%, respectively, in discriminating between metastatic (*n* = 86) and non-metastatic LNs (*n* = 49) of lung cancer, and concluded that Ki with high specificity provided a complementary value to SUVmax [8]. The study performed by Ye Q et al. strongly indicated that Ki from dynamic PET can provide superior discrimination between benign and malignant lung nodules than SUV [43]. The current study showed that the Ki-65 min, Ki-30 min, and SUVmax cut-off values for distinguishing malignant from benign lung lesions were 0.022 ml/g/min (AUC of 0.816, sensitivity of 66.40%, and specificity of 83.30%), 0.018 ml/g/min (AUC of 0.816, sensitivity of 69.50% and specificity of 83.30%), and 9.65(AUC of 0.709, sensitivity of 64.10% and specificity of 72.20%), respectively. In concordance with previous work, our study showed that both Ki-65 min and Ki-30 min have higher diagnostic efficacy than SUVmax, especially in specificity (Fig. 5). Furthermore, our study also revealed that Ki-65 min and Ki-30 min have similar diagnostic efficacy in lung lesions. In clinical practice, we preferred to improve the specificity of ^18^F-FDG PET/CT in the diagnosis of lung lesions by measuring Ki values, given that most lung cancer often shows high SUVmax values in static ^18^F-FDG PET/CT images which gives considerable diagnostic sensitivity of 87-100%, and slightly lower specificity of 50-88% with a cut-off value of 2.5 [44]. So, this potential improvement in specificity may support the use of Ki-65 min and Ki-30 min in the differential diagnosis between benign and malignant lung lesions and is a valuable addition to static ^18^F-FDG PET/CT. Such observation will require careful validation in the future. These results also further illustrated the feasibility of 30-min dynamic scanning in terms of quantitative dynamic parameters.

In the previous study of the correlation between PET/CT metabolic parameters and PD-L1 expression, it was indicated that SUVmax, total lesion glycolysis, standard uptake value ratio of PD-L1 positivity were higher than PD-L1 negativity [31–34], and the multivariate analysis revealed that only SUVmax as an independent predictor of PD-L1 positivity, and the best SUVmax cut-off value was determined to be 12.5 with sensitivity and specificity of 65.4% and 86.7%, respectively [34]. Our study also showed that PD-L1 status of NSCLC could be predicted by SUVmax at the cut-off value of 9.55 with sensitivity and specificity of 79.50% and 64.70%, respectively. In addition, the results of the present study showed that both Ki-65 min and Ki-30 min could predict PD-L1 status of NSCLC and both had the same predictive performance compared with SUVmax. Chang et al. showed PD-L1 positivity could increase the expression of glycolysis enzymes and promote the utilization of glucose in tumor [45]. Thus, for NSCLC, it may explain why SUVmax, Ki-65 min, and Ki-30 min all can predict PD-L1 expression. More importantly, the results of the present study revealed that Ki-30 min has the similar predictive power as Ki-65 min in PD-L1 expression. Therefore, the dynamic quantitative parameters obtained from a 30-min dynamic scan are sufficient to meet the clinical needs.

Interestingly, we also observed the differences in Ki-30 min and Ki-65 min between the lesion’s d_max_ in different groups. Compared with the group of d_max_<1.5 cm and 1.5 cm ≤ d_max_<3.0 cm, the group of d_max_≥3 cm had a stronger correlation between Ki-30 min and Ki-65 min, with a lower D%. While the difference between the group of d_max_<1.5 cm and 1.5 cm ≤ d_max_<3.0 cm in D% was not found. PVE could introduce large quantitative bias, especially in lesions with diameters less than 3 times the resolution of the imaging system. And PVE is affected by noise and strongly depends on the size of the lesion. The smaller the lesion, the greater the underestimation of the uptake value. Previous studies have revealed that lesions larger than 2.8 cm, are weakly or not at all affected by PVE [40]. The kinetic analyses also are subject to the PVE, which varied over time due to blood pool activity and changing tumor contrast [40, 46]. In the Ki-30 min image, the lesions of d_max_<3.0 cm might be more severely affected by PVE because of high image noise, high blood pool background uptake, and low tumor uptake. This might lead to the difference in Ki-30 min and Ki-65 min between the different groups of the lesion’s d_max_ but needs further research.

The main limitations of our study are: (i) Only chest dynamic scanning was performed, and the dynamic quantitative parameter Ki value of lung lesions was analyzed. (ii) Motion correction was not performed. (iii) Input function was not derived from blood samples. (iv) A considerable but imbalanced number of benign and malignant lung lesions were included in the study, which may lead to a statistical bias. Further study with more lesions is needed.

## Conclusion

This study indicates that an early 30-minute dynamic FDG-PET acquisition appears to be sufficient to provide good quality quantitative images and accurate dynamic parameter-Ki for quantitative assessment of lung lesions and prediction PD-L1 expression of NSCLC. Protocols with a shortened 30-minute acquisition time may be considered for patients who have difficulty with prolonged acquisitions because of it being more time-saving, patient-friendly, and without sacrificing accurate quantitative parameters.

## Data Availability

The datasets used or analyzed during the current study are available from the corresponding author on reasonable request.

## Abbreviations

^18^F-FDG: 2-deoxy-2-[^18^F]fluoro-D-glucose
AC: Adenocarcinoma
AUC: Area under the curve
BSREM: Block sequential regularized expectation maximization
D%: Percentage difference
d_max_: Maximum diameter
ICIs: Immune checkpoint inhibitors
IDIF: Image-derived input function
IF: Input function
IHC: Immunohistochemistry
Ki-30 min: Ki of the first 30 min post-injection
Ki-65 min: Ki of the first 65 min post-injection
LNs: Lymph nodes
MR_glu_: Metabolic rate of glucose
NCCN: National comprehensive cancer network
NSCLC: Non-small cell lung cancer
OS: Overall survival
PBIF: Population-based input function
PD-L1: Programmed death-ligand 1
PET: Positron emission tomography
PFS: Progression-free survival
PVE: Partial-volume effect
ROC: Receiver operating characteristic
SCC: Small cell lung cancer
SCC: Squamous cell carcinoma
SUVmax: Maximum standard uptake value
SUVmean: Mean standard uptake value
SUVs: Standard uptake values
TPS: Tumor proportion score
VOI: Volume of interest
